# Exploring religious, spiritual, traditional, and folk healing practices for oncological disorders

**DOI:** 10.1186/s12906-025-04818-w

**Published:** 2025-07-09

**Authors:** Nabila Anwar, Salma Amin Rattani, Salima Shams, Farida Bibi Mughal, Sahrish Jalaluddin

**Affiliations:** School of Nursing and Midwifery, Aga Khan University, Stadium Road, Karachi, Pakistan

**Keywords:** People psychology or preferences, Traditional, Religious, Spiritual, Folk, And healing practices, Oncological disorders, Family fear, And family pressure

## Abstract

**Objective:**

To investigate why cancer patients and their families prefer traditional, spiritual, folk, and religious healer practices over medical treatment, and to explore the various traditional, spiritual, folk, and religious healing practices used to treat oncological disorders.

**Methodology:**

A descriptive qualitative study design was used. The participants were patients diagnosed with cancer (*n* = 4), their family members (*n* = 4), and the healers (*n* = 4) providing care to these patients. In total, there were twelve participants in this research. A semi-structured, open-ended interview guide was used for the data collection.

**Study setting:**

Research was conducted in Murree, Kotli Sattian, Rawalpindi District of Punjab, Pakistan.

**Results:**

Data analysis of this study showed that factors influencing decision making for the healing practices were resources, such as unavailability of healthcare facilities within the vicinity, distances and limited finances, fear related to a new diagnosis, and cultural influence, including spiritual and religious factors. Furthermore, the types of practices performed by the patients and used by healers to treat oncological disorders were found to be religious, spiritual, traditional, and folk healing practices.

**Conclusion:**

People prefer religious, spiritual, traditional, and folk healing practices for oncological disorders for multiple reasons including cultural belief, lack of resources, inaccessibility of healthcare facilities, and lack of awareness. People use various healing practices that they believe can aid in healing, but this is due to their ignorance. These and many other factors lead to a delay in cancer diagnosis, and it is too late to save their lives. However, religious and spiritual practices can help in enhancing the quality of life when an individual is in a challenging health condition.

**Supplementary Information:**

The online version contains supplementary material available at 10.1186/s12906-025-04818-w.

## Background

Cancer is one of the leading causes of death globally [1]. There were close to 20 million new cases of cancer in the year 2022 alongside 9.7 million deaths from cancer [2]. By 2040, the global cancer burden is expected to grow to 27.5 million new cancer cases and 16.3 million cancer deaths [3]. In high income countries (HICs) cancer is a particularly common cause of death where other causes of death such as infectious diseases and maternal mortality have been significantly reduced [4]. In low and middle-income countries (LMICs) cancer related morbidity and mortality is high due to rising population, exposure to risk factors [5]. In Pakistan an LMIC, these high rates are contributed by late diagnosis [6, 7]. This delay is due to ignorance of disease, fear of cost anticipated treatment, consulting traditional and spiritual healers [6, 8]. These healers are being recommended by friends and family [6–10]. These are spiritual healers [10], faith healers using supernatural forces [11], and there are healers using different approaches and there are healers who use complementary and alternative medicine therapies [10].

Spiritual healers use holy books. Muslim believe that Allah Almighty (God) sent down divine holy books for the guidance of mankind on different Prophets to convey the message of Allah to mankind. All of these divine holy books contained nothing but the truth of their time. The Quran is the fourth sacred holy book [12]. Recitation of Quran verses decrease cell proliferation. The verses of the Quran have a beneficial effect on a person’s soul, bringing calmness and peace, which can enhance physical health and decrease the growth of cancer cells [13, 14]. The physical health of patients with cancer diagnosis is correlated with their level of spirituality and religion, with higher levels of these factors being linked to better reported physical health [15].

Type of unconventional methods that are used include traditional Greek herbal medicines, homeopathy, massage and relaxation, megavitamins, special diets and supplements and folk remedies [16]. In the chemical components of some plants like Berberis aristata (sumblo) have been shown in clinical and experimental research to have a variety of therapeutic properties, including anti-diabetic, anti-microbial, anti-cancer, antipyretic, hepatoprotective, ophthalmic, and cardiotonic activity [17]. Whereas Neem (Azadirachta indica) plant is a rich source of antioxidant activity. It is used to cure cancer. The proliferation of Hela cervical cancer cells is inhibited by using the neem seed oil. The nimbolide interferes with Hela cell cycle progression and stops it from replicating [18]. Commiphora mukul (Guggul) extract, is also effective in fighting cancers. Guggul aids in enhancing the mobility of white blood cells and enhancing the generation of red blood cells [19].

## Rationale of the study

In their clinical practice the researchers have noted that patients with cancer diagnosis before reaching medical care approach different healers for treatment. This decision is mostly influenced by their family members and other relatives, availability of traditional healers and fear of cancer. Thus, resulting in reaching to medical care at the end stage of life. Hence, cancer is being considered as death bed. Whereas, if diagnosed at an early stage, treatment is likely to work best. This research aimed at identifying the circumstances that influence cancer patients’, their family members’ decisions to seek traditional, folk, spiritual, and religious healing techniques. Also, to know about these techniques or approaches that these healers use.

### Significance of the study

Evidence-based practice is essential for health care providers and patients as well for the improvement of the healthcare setting. Therefore, it was considered to understand cancer patients’ and their families’ perspectives regarding traditional, spiritual, religious, and folk healers and the impact of these perceptions over their health. The information obtained can be useful in offering quality cancer care.

### Research question

- What are the perceptions and knowledge of patients with cancer diagnosis, their family members, and healers about traditional, spiritual, folk, and religious practices for oncological disorders?
- What are the traditional, spiritual, folk, and religious practices that are most commonly used to treat cancer patients in Murree Kotli Sattian, Rawalpindi District of Punjab, Pakistan?
- Why do people prefer traditional, spiritual, religious, and folk healers for oncological disorders?

### Objectives of the study

1. To investigate why cancer patients and their families prefer traditional, spiritual, folk, and religious healer practices over medical treatment.
2. To explore the various traditional, spiritual, folk, and religious healer practices used to treat oncological disorders.

### Study design

In this research the descriptive qualitative study design was used.

### Study population and setting

The study population included traditional, spiritual, religious, and folk healers who were treating patients with cancer diagnosis, the patients with cancer diagnosis who were receiving therapy from these healers, and the family members of these patients. Research was conducted in Murree, Kotli Sattian, Rawalpindi District of Punjab, Pakistan.

### Sampling technique

Snowball sampling technique was used to collect the data.

### Sample size

A total of twelve participants were enrolled in this study. They were four oncological patients, four family members, and four healers. In Pakistan, family members motivate cancer patients for seeking treatment from traditional healers [20]. Therefore, they were included in this research. Also, healers were included to know healing practices they use for cancer patients. These participants’ in-depth interviews were conducted till the data saturation was achieved.

### Method of data collection

A semi structured interview guide was used for data collection. Each interview was planned for 25–35 min. An audio recorder and paper pen were used to record the data. The researcher conducted interviews in the participant’s language (Urdu).

#### Pilot testing

Prior to actual data collection, pilot testing was conducted to confirm the accuracy of the interview guide.

### Ethical consideration and study duration

The study was conducted from May 2022 to December 2022 after approval from the Ethical Review Committee (ERC) of the Aga Khan University Pakistan. ERC# 2022-7223-21414.

### Inclusion criteria

- Patients with cancer seeking treatment/ therapy from traditional, spiritual, religious, or folk healers.
- Those Healers, who were currently treating patients with cancer diagnosis and were mentioned by participants.
- Family members of patients with cancer diagnosis.
- Patients receiving therapy with traditional, spiritual, religious, and folk healers, along with medical cancer treatment.
- Ages of 18–80 years.

### Exclusion criteria

- Those who had been diagnosed with illnesses or disorders other than cancer.
- Patients were diagnosed with cancer but were receiving only medical treatments.
- Individuals who refuse to participate.

### Data analysis procedure

Data analysis started concurrently with data collection and comprised a reflexive examination of the data. Tabular form was used to portray demographic data. Transcription was done using the recorded interviews. All the twelve transcripts were considered as one data set. The researcher read each transcript data segment numerous times, which led to the creation of a number of categories and themes.

### Study rigor

It is critical for researchers to ensure rigor (credibility, reliability, conformability, and transferability) and the trustworthiness of conclusions derived from qualitative research.

### Demographic data

Participants’ demographic data is presented in tables, Table 1. Family members data, Table 2. Healers Data, and Table 3. Patient with Cancer Diagnosis.

**Table 1:** Family members’ data

Family Member	Age	Marital Status	*Qualification	Type of Cancer	Relationship with Patient
F1	25–35 years	Married	Bachelors	Liver cancer	Daughter
F2	25–35 years	Married	Middle	Breast cancer	Son
F3	25–35 years	Unmarried	Matric	Skin cancer	Daughter
F4	25–30 years	Married	Master + hafiz-e-Quran	Blood cancer	Daughter

**Table 2:** Healers’ data

Healers	Age	Marital status	*Qualification
H1	45–55 years	Married	Primary
H2	55–65 years	Married	Matric
H3	35–45 years	Married	Intermediate, Dar-e-Nazami, Hafiz-e-Quran
H4	55–65 years	Married	Primary + study in Amuliyaat

**Table 3:** Patient with cancer diagnosis data

Patient	Age	Marital Status	*Qualification	Type of Cancer
**P1**	25–35 years	Married	Graduation	Blood cancer
**P2**	75–85 years	Married	Middle	Skin cancer
**P3**	55–65 years	Married	Primary	Axillary lymph node carcinoma
**P4**	55–65 years	Married	Middle	Chronic lymphocytic leukemia

*Description for qualification: Primary means schooling till 5th grade,* middle means 8th*,* matriculation means 10th*,* intermediate means 12th grade and graduations means 14 years of schooling or education*

### Themes and categories

Themes and categories are presented in Table 4 followed by the descriptions supported through participants’ quotes during interviews.

**Table 4:** Themes and categories

Theme	Categories	Subcategories
**1.** Factors influencing decision-making for the healing practices	Resources	Unavailability of healthcare facilities within the vicinity
		Limited finance
		Distance
	Fear related to a new diagnosis	
	Cultural influences	Religious
		Spiritual
**2.** Type of practices performed by patients and healers used to treat oncological disorders	Religious healing practices	
	Spiritual healing practices	
	Traditional and folk healing practices	

### Factors influencing decision making for the healing practices

Cancer patients and their families prefer traditional, spiritual, folk, and religious healers over medical treatment. This means that they first go to these healers for the treatment of cancer because they have a strong belief in their healing practices. Other reasons why they prefer these practices include cultural belief system, lack of resources, fear of cancer diagnosis, influences from family, friends, and relatives, and considering this treatment cheaper than the medical treatment.

#### Resources

Lack of resources is one of the main reasons for selecting the easiest method of cancer treatment.

#### Unavailability of health care facilities within the vicinity

There was a government hospital in the area, but it was too far away. There were secondary hospitals, but there was no facility for screening for cancer. The lack of resources and inaccessibility of health care facilities in the area was also a contributing factor to cancer patients’ and their families’ preferences for spiritual, religious, folk, and traditional healing therapies.

One of the family members said, “Our biggest problem was that we did not have enough money to treat cancer. There was no government hospital nearby; we had to go to Rawalpindi for treatment”. (F3)

#### Limited finance

One of the factors influencing is the low cost of treatment. Most people are unable to afford medical treatment due to high expenses. According to a folk healer, “There are poor people in the hilly area, so they cannot go far for treatment, so we use these herbs for medicine and provide them free of cost”. (H1)

#### Distance

One of the most pressing issues that people face is the long distance.

One patient said,

“The shrine in our village is very close by, and we can quickly go there without paying for transport. Villagers do not have enough money to pay for transportation to travel to another city. That is why our priority is spiritual treatment and shrine”. (P2)

#### Fear related to new diagnosis

People’s fears about cancer or any other disease also contributed to their preferences in selecting these healing practices from healers. One family member expressed, “My mother was afraid about the diagnosis of cancer or any other disease that was not affordable for us”. (F4)

Fear related to any disease is a natural thing, but when participants are unable to cope with their challenges, it causes a problem. One positive thing in the current study was that participants were not anxious about stigmatization related to cancer because their families were cooperative. However, many other factors create a fear in their minds, preventing them from mentioning their signs and symptoms in front of their families. These fears resulted in a delay in cancer diagnosis and management.

#### Cultural influences: religious and spiritual

It is a cultural belief that spiritual, religious, traditional, and folk healers should be approached first for disease treatment because many people who go to these healers get healed. One participant said, “If we had enough resources, we could go for medical treatment first and Dum (recitation of the Holy Quran) could also be offered”. (F1)

One participant said that “We were told by our elders and our forefathers that we should go on pilgrimage. That is why we trusted them”. (F2)

The researcher also observed that many of the participants go for other healing practices for the sake of their elders’ satisfaction and for respecting their views. Even though they know that medical treatment is better than other practices, due to varying circumstances, they decide to get benefit from other therapies.

### Type of practices performed by patients and used by healers to treat oncological disorders

People prefer different traditional, folk, spiritual, and religious healing practices for oncological disorders. They utilize different practices like herbs, amulets (taweez), holy water, dum water, rolling of shrine stones, and visiting the shrine for healing purposes.

#### Religious healing practices

People prefer religious practices of healing purposes because they have a strong belief in them. During discussion, one male participant told the researcher, “We can treat cancer by consulting a doctor, but we may also go for religious activities like Dum, Darood, and spiritual and religious practices. These treatments are better”. (F3)

Another female shared about her mother’s treatment practices, saying,

She goes for Dum and Taweez. She takes taweez stirs the milk with it and sometimes the water. She also wears a taweez (amulet) around her neck. (F4)

Another religious healer explained, “There are many verses in Quran, like the recitation of Ayat-ul-Kursi twenty-one times for twenty-one days, which is recommended for cancer patients”. (H4)

The participant described it further and said, “Magic [jadoo] is also one of the causes of cancer or many other diseases. It is important that the magic should also be treated, and then the patient can be healed”. (H4)

#### Spiritual healing practices

In every culture, people utilize different spiritual healing practices. People engage in spiritual healing practices for the purpose of physical and mental health and wellness, comfort, and healing. A family member explained,

“Our elders believe in a holy site (mazaar) in our village, which we call Khan Ghah. There are some special stones on the holy site, and people take the stone and roll it on the affected part of the body (where the cancer is) for healing purposes”. (F1)

#### Traditional and folk healing practices

In Pakistan, different folk healing practices are used for the management of cancer. The researcher noticed that the trends of folk healing practices are common in Murree Kotli Sattian. There the People utilize different herbs to cure different diseases, and cancer is one of them. These herbs are present in the mountainous area.

During a conversation with a folk healer, he said,

“I use different types of herbs for cancer treatment. Their names are Khoor, Sumbal, Goglo, black jeery, and Meda saak. Their taste is bitter. When we collect herbs, these are wet, then these are dried and used for several types of cancer, ulcers, and other diseases. Khoor herb is special for cancer patients. People use this herbal medicine until they recover from cancer”. (H02)

During data collection, the researcher noticed that most people were using folk and traditional healing practices for cancer management, but very few of them knew the names of the herbs. Healers practically use these medications on patients, and they get better, the healers think that these herbs are good for that specific disease.

## Discussion

### Factors influencing decision making for the healing practices

Patients with cancer diagnosis, and their families prefer traditional, spiritual, folk, and religious healers over medical treatment. There are many other reasons why the cancer patients and their family prefer these practices, such as: cultural belief system, lack of resources, inaccessibility of health facility, long travel, fear of diagnosis, paternalistic approach of family members, and relatives, and cheaper treatment.

About 80% of the world’s cancer patients reside in lower- and middle-income countries (LMICs), but only 5% funding for cancer research worldwide is directed toward this disease [15]. Patients in LMICs face difficulties due to limited access to primary prevention strategies resulting in delayed diagnosis [15].

It is reported that among the women with breast cancer who participated in research in Pakistan, 69% were in the third and fourth stages when they for the first time presented for medical treatment and of these women 39% belonged to the northern areas of Pakistan [11].

#### Resources

Where resources are concerned, no health care facility was found near the vicinity in the setting selected for this research. Hence, people seldom see a doctor, due to large distances and the high expense of medical treatment. These findings are reported in another research [17, 21].

Women from low-income families usually avoid going to hospitals and instead treat their illnesses with Holy water and taweez (amulet). Illiteracy (no formal education), low socioeconomic status, and incorrect perceptions about sickness and screening also play a role. Moreover, generally, the people who contact traditional healers are more likely to be illiterate [21–23].

On the one hand, for patients suffering with cancer, its treatment is beyond their budget. Besides, the health care providers’ bad behavior can create fear in the mind. Therefore, to improve patients’ compliance and quality of life, it is also necessary to address the psychosocial component, i.e., emotional stress, that patients experience after a cancer diagnosis [24].

The healthcare industry is responsible for the delays due to poor service quality, unprofessional behavior on the part of doctors, and lengthy wait times at public health centers [21]. Moreover, a mismatch between patient load and the infrastructure is one of the causes for treatment delay in hospitals [25].

One particular problem that was noticed in this study was that when patients have a symptom, they go to a small clinic or near-by hospital, where they symptomatically treat the patient without diagnosing the disease. Hence, healthcare practitioners’ ignorance, misleading assurances, and lack of competency of health care specialists in diagnosis led to be delayed identification of cancer [6, 21, 26]. To ensure effective therapy, a positive outcome, and an enhanced quality of life, early identification, within three months of seeing signs, is absolutely crucial [24, 27].

#### Fear related to new diagnosis

People’s fears about cancer or any other sickness may be a contributing factor in choices of different healing modalities, traditional, spiritual, folk, and religious healers. Mostly, their patients hide their early symptoms due to fear of disease diagnosis. Hence, patients’ misconceptions about the condition, perceived stigma, anxiety, and denial of oncological disorders are a few of the factors delaying the patient’s presentation of symptoms to a healthcare practitioner [6, 24, 28, 29].

One of the reasons that contributes to delay in the presentation of breast cancer is the cultural aspect, which hinders women from reporting issues related to private body parts; opposite gender health care professionals further delay female patients. Fear related to cancer and its treatment also contribute to delay in diagnosis [27].

In the present study the researcher noticed that when participants received a diagnosis of cancer, their family members were supportive. It was noted that the family members, within their capacity, did everything for the patient’s treatment but due to the late diagnosis, they lost their beloved ones.

#### Cultural influence: religious and spiritual influence

Participants’ descriptions indicated that it is a cultural belief that spiritual, religious, traditional, and folk healers should be approached first for the treatment, because many people go to them and get healed. Moreover, in the Pakistani culture, people obey their elders’ orders with respect, and then their cultural practices run from generation to generation. Hence, culture influenced the participants’ decision for obtaining these healing practices.

Most of the people reported that they used traditional practices to get rid of the side effects of conventional medications. Also, family and friends recommended using these therapies [30]. In many cases, culture may have had an even bigger influence on management than core religious beliefs [31]. This is because cultural values and beliefs play an important role in accessing healers when a patient is suffering from cancer, and their family, and friends support them in accessing the healer and paying the healer [6, 27]. Family bonds with husband and father are strong in Asian countries, and their decisions have a strong influence on family decisions, particularly with regard to women [23, 27, 29].

Breast cancer screening for Muslim women may be impacted by cultural beliefs, attitudes, and awareness. Moreover, it can be affected by religion, stigma, and health attitudes, which can also delay detection and treatment [10, 26, 29]. Lack of health care systems and infrastructure, as per cultural requirements, cause hindrance in accessing and utilizing of health care facilities [27]. A study in Pakistan enrolled 241 cancer patients of these almost 60% believed that by engaging in specific religious rites, they would be able to cure their disease [32].

### Type of practices performed by patients and used by healers to treat oncological disorders

Many people believe that they can heal their diseases with other healing practices. Like in this study, most participants utilized different religious, spiritual, traditional, and folk healing practices to cure cancer. Patients felt better symptomatically, but their cancer disease got worse, and then they approached doctors for medical treatment.

#### Religious and spiritual healing

Religion is a multifaceted concept that includes beliefs, customs, and rituals pertaining to the transcendent, i.e., God. Religious belief is frequently viewed as a shared, institutionalized religion, with unique rituals and customs. Spirituality is a unique and individual aspect of religious experience, whereas religion typically conveys a fixed collection of concepts [33].

The religious healers used two methods for healing. One is the application of the Holy Quran for healing, and the second is giving instruction to people when they have health issues [11]. To manage their illnesses, people go to spiritual healers and the shrine. The ability to find comfort in religion and spirituality has proven crucial in helping people deal with the difficulties of diagnosis, treatment, and survivorship [33, 34].

In this study, some participants were unaware of the disease, and people visited holy places and rolled stones on their bodies for healing. Such people may adopt spiritual and traditional healing. Furthermore, many religious people strongly believe that supernatural power can heal illnesses [23]. Further, it has been discovered that engaging in prayers, Ruqyah (refers to healing methods using Quranic verses), and recitation of Quran reduces the likelihood of developing depression [35].

According to a study, the recitation of Quran verses decreases cell proliferation. The verses of the Quran have a beneficial effect on a person’s soul, bringing calmness and peace, which can enhance physical health and decrease the growth of cancer cells [36]. This is based on narrations that the Prophet PBUH and his companions practicing ruqyah, treating others by reciting surah al-Fathia. Moreover, listening to the Holy Quran is a form of music therapy that aids in the release of endorphins, which lowers patient anxiety and depressive symptoms, and it enhances excellence of life [35].

Patients with cancer diagnosis have a strong belief that religion and spirituality may cure cancer. Thus, individuals’ faith and religious practices are seen as a sign of inspiration and a means of coping with terrible illnesses [23]. The physical health of patients with cancer diagnosis is correlated with their level of spirituality and religion, with higher levels of these factors being linked to better reported physical health [37]. In this study all the participants were Muslims and they said they strongly adhered to Islamic principles. One of the fundamental principles of Islam is the belief in fate and destiny.

Some patients burned incense sticks in their room. Many patients employed meditation, yoga, and faith-healing activities to get psychologically refreshed and relieve symptoms, including tension, emotional discomfort, nausea, and pain [38]. These techniques promote stress reduction, a sense of well-being, and enhanced excellence of life expectancy in patients recently diagnosed with cancer [15]. Faith healers offer a variety of amulets (Taweez), providing relief through meditating with incense sticks and other such objects in an effort to reduce distress signs [11].

#### Traditional and folk healing

Many individuals use various types of traditional and folk healing methods for cancer treatment. There are cultural practices that run from family to family. In Pakistan, especially, in the northern areas, there are many types of herbal plants that help people use to cure illnesses. Traditional and folk healers use different types of herbs for many diseases, but for the cure of cancer, they used Berberis Aristata (sumblo), Commiphora mukul (gugglo), Litsea chinensis (medasaka), Black Cumin Seeds (kali jeeri), and Azadirachta indica (Neem or khoor boti).

The World Health Organization estimates that 80% of the people worldwide utilize herbal remedies to treat a variety of ailments [39]. There are close to 6,000 wild species of flora, 600 of which have been recorded as being utilized in traditional medicines, and there are roughly 50,000 certified folk medicine practitioners in Pakistan [40]. In Pakistan’s rural areas, almost 80% of the population mainly relies on herbal remedies [26].

The chemical components of some plants like Berberis aristata (sumblo) have a variety of therapeutic properties, including anti-diabetic, anti-microbial, anti-cancer, and cardiotonic activity [41, 42]. The anticancer properties of the stems of B. Aristata have cytotoxic impact of the methanolic extract on the cell growth activity in the MCH-7 breast cancer cell line [18]. Due to the inclusion of several alkaloids, B. Aristata not only prevents cancer from proliferating, invading, and metastasizing but also improves the outcomes of chemo radiotherapeutic therapy [42].

Neem (Azadirachta indica) plant is a rich source of antioxidant activity. It is used to cure cancer [18]. Furthermore, neem components play a significant role in cancer prevention because of their ability to modify the tumor environment through a variety of processes, such as reducing angiogenesis and increasing cell toxicity [43]. Additionally, the immunomodulatory properties of many neem constituents suggest that this plant has unexplored potential as a prophylactic vaccine in the future [44].

Litsea Glutinosa (Medasaka) is also used for cancer treatment. The traditional therapeutic benefits of L. glutinosa (Medasaka) for cancer treatment are supported scientifically by its antioxidant and anticancer properties [45]. A chromone alkaloid found in L. glutinosa exhibits a wide range of biological actions. There include anti-cancer, anti-hyperlipidemia, anti-inflammatory, and anti-inflammatory actions [46].

Commiphora mukul (Guggul) extract, is also effective in fighting cancers. Guggul aids in enhancing the mobility of white blood c.ells and enhancing the generation of red blood cells [47]. The extract obtained from C. Mukul has antibacterial and antifungal activities that prevent wound infection [48].

Black Cumin Seeds (Kali Jeeri) have been used for more than 2000 years. Black cumin seeds are referred to as “Habbatul Barakah” in Arabic, which corresponds to “the seed of grace.” According to a Prophetic (S.A.W) hadith, black seed is a cure-all for all ailments except death, hence it is regarded in Islam as one of the most potent forms of healing medication [33].

Studies have shown the effects of herbs. These herbal plants can be used to treat cancer and a variety of other illnesses. Plants are a blessing from God. For cancer treatment, scientific exploration with respect to utilization of such herbs is needed.

### Implications for policy and practice

- It is utmost important that the government should provide screening facilities for the early detection of cancer.
- Although there are many hospitals available for screening, they do not have enough resources, so healthcare policymakers should check those hospitals and provide the required resources.
- There are many herbal plants available in the northern areas of Pakistan that have anticancer properties. Research is important to recognize these herbs in prevention and treatment of cancer.
- Mass campaigns are required to raise awareness about early diagnosis and management of the disease.

### Limitations

This study was conducted at a single site; thus, one limitation is that it cannot be generalized to the broader population. Moreover, the study was carried out in a rural region, so the findings might not apply to Pakistan’s urban areas.

## Conclusion of study

People prefer religious, spiritual, traditional, and folk healing practices for oncological disorders for multiple reasons, like cultural belief, lack of resources, inaccessibility of healthcare facilities, and lack of awareness. People use various healing practices that they believe can aid in healing, but this is due to their ignorance. These and many other factors lead to a delay in cancer diagnosis, until it is too late to save their lives. Even when people agree to medical treatment, due to their financial problems, they are unable to afford the medical treatment. Moreover, they need to understand that some of the other healing practices are safe, can be continued with medical treatment. Religious and spiritual practices can also help in enhancing the quality of life when an individual is in a challenging health condition.

## Electronic supplementary material

Below is the link to the electronic supplementary material.

Supplementary Material 1

## Data Availability

The datasets generated and analyzed during the current study are not publicly available because I generated themes from the data, and I have obtained consent from the participants that we will not share your data directly. All data is available, and upon request, the corresponding author, Miss Nabila Anwar, can email: nabila.anwar22@alumni.aku.edu will shared.
