# Supplementary material for: Exploring religious, spiritual, traditional, and folk healing practices for oncological disorders
Source: BMC Complement Med Ther. 2025 Jul 9;25:252. doi: 10.1186/s12906-025-04818-w (PMC12243430; doi:10.1186/s12906-025-04818-w)
Supplement: Supplementary file 1 — Supplementary Material 1 [file 12906_2025_4818_MOESM1_ESM.pdf]

**Appendix D**

**Interview Guide**

**TITLE:** Peoples' preferences for traditional, spiritual, religious, and folk healers for oncological disorders.

**Section: 1**

**Demographic Data:**

**Gender:**

- Male
- Female

**Age in years:**

- _______ years

**Marital Status:**

- Single
- Married
- Separated/Divorced
- Widowed

**Education status:**

- Primary
- Middle
- Matric
- Intermediate
- Graduation
- Master
- PhD
- Others ____________________

**Section: 2**

**For oncological patients:**

1. How long have your patient been diagnosed with cancer?
2. What do you know about oncological disorders?
3. What are the causes of oncological disorders?
4. How can we treat oncological disorders?
5. Do you think that medical treatment is better to deal with oncological disorders than traditional, spiritual, folk, and religious healing?
6. Why do you favor traditional, spiritual, religious, and folk healers in the treatment of oncological disorders?

7. What are the barriers that challenge you to seeking treatment from traditional, spiritual, religious, and folk healers?

8. What do you think if I say that traditional, spiritual, religious, and folk healers should continue their practice with medical treatment?

9. Can you tell me about the practices you use to treat the oncological disorders that your healers have told you about?

10. Who told you about these (traditional, spiritual, religious, and folk) healers?

11. Are you satisfied with (traditional, spiritual, religious, and folk) healing practices for your cancer treatment?

**Appendix: E**

**Interview guide**

**For Family Member**

1. How long has your patient been diagnosed with cancer?

2. What do you know about oncological disorders?

3. What are the causes of oncological disorders?

4. How can we treat oncological disorders?

5. Do you think that medical treatment is better to deal with oncological disorders than traditional, spiritual, folk, and religious healing?

6. Why do you favor traditional, spiritual, religious, and folk healers in the treatment of oncological disorders for your patients?

7. What are the barriers that challenge you to seeking treatment from traditional, spiritual, religious, and folk healers for your patient?

8. What do you think if I say that traditional, spiritual, religious, and folk healers should continue their practice with medical treatment?

9. Can you tell me about the therapies you utilize for patients with oncological disorders that your healers have recommended?

10. Who told you about these (traditional, spiritual, religious, and folk) healers?

11. Are you satisfied with (traditional, spiritual, religious, and folk) healing practices for your cancer patients?

**Appendix: F**

**(Interview Guide for healers)**

1. How long have you been treating cancer patients?

2. What do you know about oncological disorders?

3. What are the causes of oncological disorders?

4. How can we treat oncological disorders?

5. Do you think that medical treatment is better to deal with oncological disorders than traditional, spiritual, folk, and religious healing?

6. What do you think if I say that traditional, spiritual, religious, and folk healers should continue their practice with medical treatment?

7. Can you tell me about the therapies you utilize for patients with oncological disorders?

8. Who teach you about these (traditional, spiritual, religious, and folk) therapies?

9. Are you satisfied with (traditional, spiritual, religious, and folk) healing practices for cancer patients?
